# Molecular Design Strategies to Enhance the Electroresponse of Polyelectrolyte Brushes: Effects of Charge Fraction and Chain Length Dispersity

**DOI:** 10.1021/acs.macromol.4c02579

**Published:** 2025-01-23

**Authors:** Leon A. Smook, Sissi de Beer

**Affiliations:** Department of Molecules and Materials, MESA+ Institute, University of Twente, P.O. Box 217, 7500 AE Enschede, The Netherlands

## Abstract

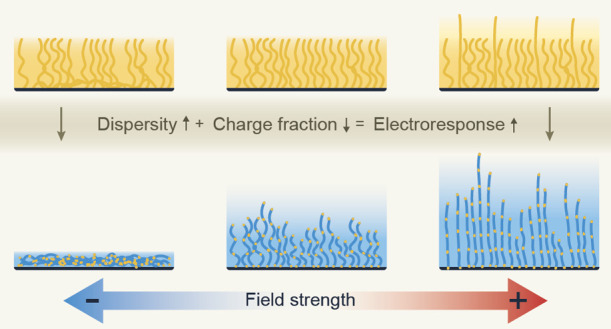

Polyelectrolyte brushes are functional surface coatings that react to external stimuli. The response of these brushes in electric fields is nearly immediate as the field acts directly on the charges in the polyion, while the response to bulk stimuli such as temperature, acidity, and ionic composition is intrinsically capped by transport limitations. However, the response of fully charged brushes is limited because large field strengths are required to achieve a response. This limits the application of these brushes to architectures such as small pores or nanojunctions because small biases can generate large field strengths over small distances. Here, we propose a design strategy that enhances the response and lowers the field strength required in these applications. Our coarse-grained simulations highlight two approaches to increase the electroresponse of polyelectrolyte brushes: dispersity in the chain length enhances the electroresponse and a reduction in the number of charged monomers does the same. With these approaches, we increase the relative brush height variation from only 28% to as much as 227% since in partially charged brushes, more chains need to respond to screen the imposed field and the longer chains in disperse brushes can reorganize over large distances. Additionally, we find that disperse brushes show a stratified response where short chains collapse first and long chains stretch first because this stratification minimizes the change in conformational energy. We envision that our insights will enable the application of electroresponsive polyelectrolyte brushes in larger architectures or in small architectures using smaller biases, which could enable a stimulus-responsive pore size modulation that could be used for filtration and molecular separations.

## Introduction

Stimulus-responsive polymer brushes transform the properties of surfaces and create systems that respond to changes in its physicochemical environment. In this manner, these brushes extend the functionality of existing systems or even enable completely novel applications through the responsive nature of polymers.^1^ To illustrate, polymer brushes have introduced thermal control over the propagation speed of self-propelled supramolecular nanomotors by restricting the fuel inlet,^2^ have enabled the development of an antibacterial wound treatment that uses magnetic, photothermal nanoparticles with a thermoresponsive brush acting as an antibactorial velcro,^3^ and can even be used to selectively separate nanoparticles by size.^4^ Polymer brushes can also change the lubrication properties of surfaces,^5^ reduce fouling of substrates,^6^ introduce antimicrobial activity,^7,8^ functionalize nanopores,^9,10^ enable new sensing mechanisms,^11,12^ or modify the interaction of brushes with air for sensing, separation, and smart adhesives.^13^ Over the last decades, polymer brushes have become increasingly accessible to the research community.^14^ These coatings of high-density surface anchored polymer chains can now even be grown in a cytocompatible manner^15^ or with extreme brush heights.^16,17^ Advances in controlled radical polymerization^18^ such as surface-initiated atom-transfer radical polymerization (SI-ATRP)^19^ allow for a wide variety of polymer brushes to be synthesized with good control over the polymer length, the grafting density of chains, and the chain length polydispersity.^20,21^ Additionally, application of brushes in nanotechnology have become more feasible with access to synthesis methods that have improved oxygen tolerance and no longer require toxic metal catalysts^22^ as well as with methods that increase the durability of the synthesized coatings.^23^

Often stimuli-responsive brushes react to changes in the environment that persist throughout the entire system, like pH and temperature. However, such stimuli need time to set and reset the properties of the brushes. The stimuli need to reach the brush from its surrounding and not all of the stimulus that is introduced in the system directly contributes to the response of the brush. Electric fields can be a more efficient trigger. If the stimulus-responsive brush contains charged monomers, an applied electric field creates physicochemical changes near the electrode causing a localized response in the structure and properties of the brush. From a practical perspective, electricity as a stimulus for polyelectrolyte brushes has also been explored. For instance, friction between brushes can be significantly reduced;^24^ cantilevers can be oscillated;^25^ the wettability of the surface can be adjusted^26^ and proteins can be captured and released on command.^27^ From a fundamental perspective, the electroresponse of polyelectrolyte brushes has been studied using theory and simulation^28−43^ as well as, to a limited extent, using experimental methods.^26,44,45^ Charged polyelectrolyte chains change their conformation in an electric field: A fraction of the chains stretch or collapse when an electric field is applied.^32,34^ With increasingly strong fields, more and more chains respond. This behavior not only holds for random copolyelectrolyte brushes, but also for copolyelectrolyte brushes with a gradient in charge along the polymer backbone.^43^ However, the response of these brushes is limited because it requires extremely large field strengths. This limitation introduces a challenge in the use of the electroresponse in applications and begs the question: How can we enhance the electroreponse of polyelectrolyte brushes and increase its application potential?

Fundamental studies usually consider perfectly monodisperse brushes, while even the most well-controlled polymerizations create brushes with some degree of dispersity in chain lengths. The properties of a polymer brush can change significantly when the dispersity changes.^46−48^ For instance, the desorption characteristics of *Staphylococcus epidermidis* from pH-responsive poly(acrylic acid) can be enhanced by introducting dispersity in the brush,^49^ the inclusion of particles in the brush can be affected,^50^ the structure of spherical polyelectrolyte brushes,^51^ or the antifouling performance of the coating.^52^ Similarly, dispersity in the side-chains of grafted bottle-brushes can significantly affect its hydration behavior.^53^ Since dispersity affects the properties of brushes, this design parameter could also influence the electroresponse.

Theoretical work on disperse uncharged polymer brushes is available in the literature (e.g., the seminal work by Milner, Witten, and Cates^54^), but the electroresponse of disperse polyelectrolyte brushes have been studied in just a few publications. Okrugin et al. studied bidisperse and tridisperse polyelectrolyte brushes with self-consistent field theory.^55^ Zhang et al. studied small, bidisperse brushes with coarse-grained molecular dynamics simulations.^56^ They found that short chains respond different to longer chains, which was confirmed by self-consistent field calculations from the same group.^57^ Yet, it is still unclear whether the observations in these perfectly bidisperse brushes translate to brushes with chain length dispersities more representative for controlled radical polymerizations.

Here, we present simulations that reveal three effects of chain length dispersity and degree of ionization that may seem counterintuitive at first glance. First, polyelectrolyte brushes become more responsive to electric fields if the polymer contains fewer charged monomers. And second, the effect of an electric stimulus on the brush height becomes stronger when the dispersity in the length of the grafted chains increases. This second finding means that tight control over the polymerization is not required for applications that rely on changes in brush height. Finally, our simulations also reveal a stratified response with respect to the chain length in disperse brushes: shorter chains collapse first, while longer chains stretch first. This stratification can have profound implications in applications where the free chain ends are postmodified with functional groups: Those groups attached to shorter chains will be more affected upon field-induced collapse, while those attached to longer chains will be more affected under field-induced stretching. We envision that our results will enable the development of new electroresponsive technologies that use polyelectrolyte brushes to introduce additional functionality.

## Model and Methods

Via coarse-grained molecular dynamics simulations, we study the electric response of polyelectrolyte brushes with a disperse chain length distribution using an implicit good solvent. We simulate our brushes using a Kremer–Grest model^58,59^ with charged beads and explicit counterions. All simulations are performed using reduced Lennard-Jones units and all results are reported in this units as well. LAMMPS has been used to run all simulations.^60,61^

### Simulation Box

Each simulation is performed in a simulation box containing 100 chains at a grafting density of 0.1 σ^–2^, giving box dimensions of 31.6 σ for each in-plane dimension. In this work, the Bjerrum length (λ_B_) is 1 σ, which in water at room temperature corresponds to approximately 0.71 nm, giving a grafting density of 0.2 nm^–2^. The box height is set to be 500 σ taller than the tallest chain in the initial configuration to prevent chains from reaching the top wall of the simulation box. A simulation box has periodic boundary conditions in the in-plane directions and fixed boundary conditions perpendicular to the grafting plane.

### Brush Architecture

In line with previous coarse-grained work,^62,63^ we model chain length dispersity using a Schulz–Zimm distribution, which has been found to be a reasonably accurate description of reversible deactivation radical polymerizations.^64^ Each brush consist of 100 chains of which the chain lengths are sampled from a Schulz–Zimm distribution which can be described as
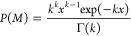
1where *k* is the shape factor of the distribution and *x* the scale of the distribution such that *x* = *M*/*M*_n_. The shape factor can be linked to the dispersity (*Đ*) of the chain length distribution such that *k* = 1/(*Đ* – 1). A variety of brushes have been simulated with values of *Đ* of 1.0, 1.2, 1.4, 2.0. Such dispersity values are common in controlled radical polymerizations that are often employed to grow polymer brushes experimentally.^18^

Chains are grafted to the grafting plane at anchor points that have been generated using a Poisson disk sampling method in line with previous work in our group^65^ with a grafting density of 0.1 σ^–2^. Each chain consist of a bonded sequence of charged (*q* = +1, *m* = 1, *r* = 1.0) and neutral monomers (*q* = 0, *m* = 1, *r* = 1.0σ). The identity of each monomer is chosen randomly such that a random copolymer with an average charge fraction ⟨*f*⟩ < 1.0 is achieved for partially ionized brushes and ⟨*f*⟩ = 1.0 for fully ionized brushes. All charged monomers are accompanied by a smaller counterion (*q* = −1, *m* = 1, *r* = 0.5σ). This counterion size corresponds to 0.36 nm in real units, which is roughly the diameter of a chloride ion.

### Interaction Potentials

The nonbonded interactions in our simulation are modeled via a truncated and shifted Lennard-Jones potential
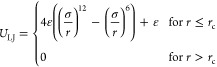
2combined with a Coulomb potential
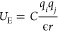
3where *r*_c_ is the cutoff distance of the Lennard-Jones potential. Here, we set *r*_c_ = 2^1/6^σ at the minimum of the Lennard-Jones potential creating a purely repulsive potential to model good solvent conditions. σ is the zero-crossing of the Lennard-Jones potential, ε is the depth of the interaction potential (here, ε = 1.0 for all particles except between counterions where ε = 0.1). Cross-interaction strengths are computed using geometric mixing rules. *q*_*i*_ and *q*_*j*_ are the charges on particles *i* and *j*, ϵ is set to 1.0, and *C* is an energy conversion constant equal to 1/(4πϵ_0_). Long-range charge interactions are computed using a particle–particle/particle–mesh algorithm with an adjustment for 2D-geometries^66,67^ and a relative accuracy of 10^–4^.

Bonded interactions are modeled using a combined finite-extensible nonlinear elastic potential and Weeks–Chandler–Anderson (WCA) potential
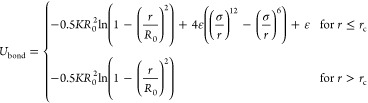
4where we use *K* = 30εσ^–2^, *R*_0_ = 1.5σ, ε = 1.0, and σ = 1.0. In melts, these parameter values have shown to prevent unphysical behavior and bond crossing.^58^

### Simulation Procedure

Each configuration starts with all chains in a fully extended state. This initial configuration is relaxed with an energy minimization. Subsequently, the brush is simulated in *NVT* with a displacement limitation of 0.05σ per time step (here d*t* = 0.005τ) with a Langevin thermostat at a reduced temperature of T* = 1.0 and a damping factor of 100 d*t* for 10^5^ time steps. Next, the displacement limitation is lifted and the simulation is continued for an additional 10^5^ time steps. Then, we impose a homogeneous electric field on the simulation box such that each charged particle experiences an additional electric force equal to
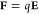
5in the direction perpendicular to the grafting plane. We continued our simulation for another 5 × 10^5^ time steps to equilibrate the brush under these conditions. We check for equilibrium by observing a lack of drift in the density profiles of the brushes and stabilization of the RMSD of the polymers during the production run (SI Section 1). Finally, we continue our simulations with a production run of between 1 × 10^6^ and 2 × 10^6^ time steps where we capture the density profiles of the charged monomers, neutral monomers, and counterions. Additionally, we capture the positions of all particles every 5 × 10^3^ times steps.

## Results

We model polyelectrolyte brushes using a coarse-grained model where the polymers are represented by a bead–spring model. The interactions between bonded beads are modeled using a FENE potential with Kremer–Grest parameters^58,59^ and the nonbonded interactions consist of a component for neutral interactions based on the Lennard-Jones potential and one for charged interactions based on the Coulomb potential (for details, see Model and Methods). Such coarse-grained models remove all chemical detail from the system, allowing us to obtain insights on the behavior of a generic polyelectrolyte brush.^68^ We present our results in reduced units, however, these units can be transformed into approximate real values. This transformation should be done with extreme caution and is only provided here to give the reader a sense of magnitude and scale compared to experimental systems. The basis unit of length in our system (σ) corresponds to approximately one Bjerrum length (0.71 nm) and the basic unit of energy (ε) corresponds to the thermal energy *k*_B_*T* (25.7 meV at room temperature). These values give that one reduced unit of electric field strength (E*) corresponds to roughly 3.6 × 10^7^ V/m. In our simulations, we expose the brushes to field strengths ranging from *E* = −15.0 E* to *E* = 15.0 E*. While the field values are rather extreme from an experimental perspective, field strengths on the order of 0.1 to 1.0 V/nm are not uncommon in simulations on polyelectrolyte brushes^69^ and can also be achieved in nanopores and across nanojuntions, for instance through a potential bias of only 1 to 10 V across a 10 nm junction.

All systems studied here consist of a similar amount of monomer, but the chain length dispersity varies from *Đ* = 1.0 (perfectly monodisperse) to *Đ* = 2.0 (very disperse) in line with dispersities commonly encountered in surface-initiated controlled radical polymerizations.^18^ Additionally, we study systems that are fully ionized (i.e., all monomers carry a charge) or partially ionized. This latter case could be seen as a pH-responsive polyelectrolyte that has partially dissociated or as a copolymer of neutral and charged monomers. We systematically vary the charge fraction in the brushes to observe trends in the electroresponse of these systems. We find an optimal response for a charge fraction around 14%. With these approaches, we increase the relative brush height variation from only 28% to as much as 227%.

### Chain Length Dispersity Affects the Structure of Unperturbed Brushes

**Figure 1:**
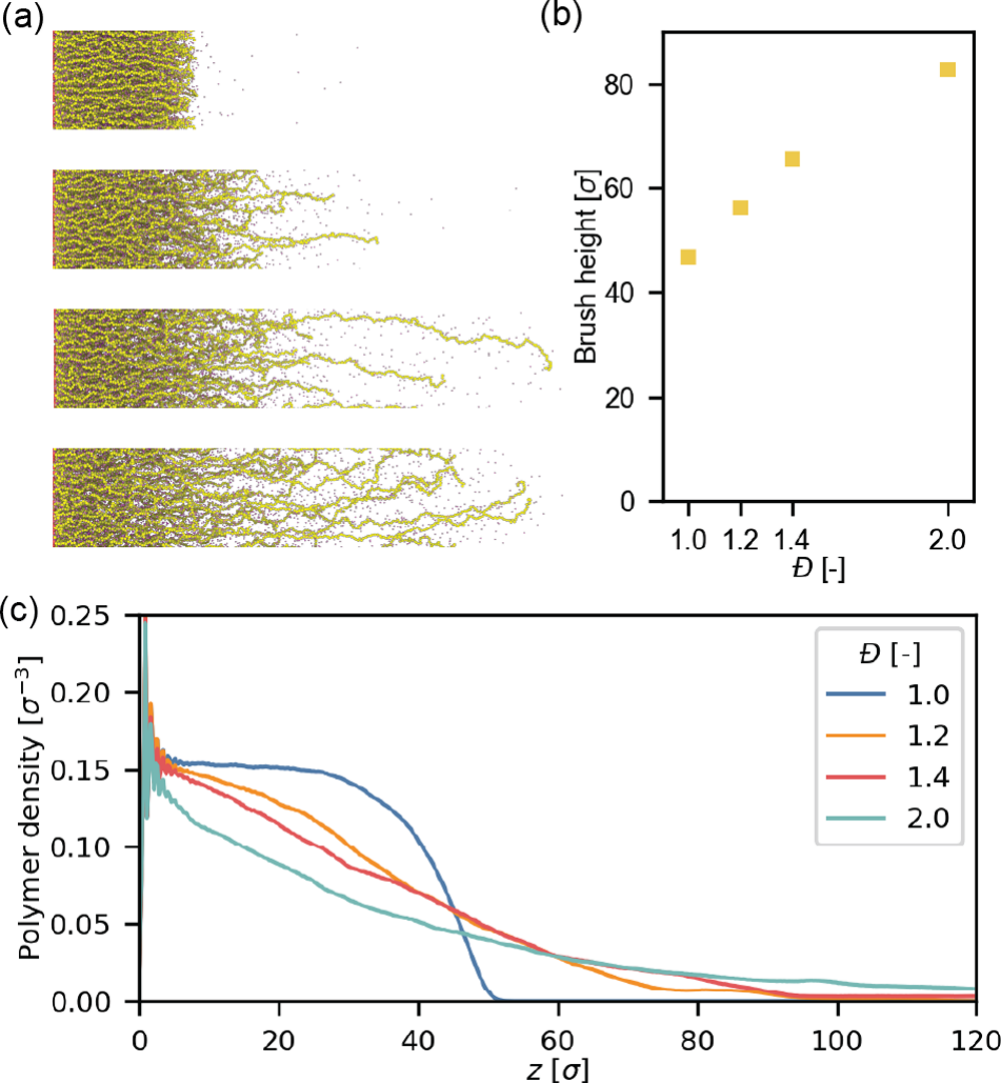
Effect of chain length dispersity on the equilibrium properties of polyelectrolyte brushes with a grafting density of 0.1σ^–2^ and a charge fraction ⟨*f*⟩ = 1.0. (a) Snapshots of the coarse-grained model. (b) Equilibrium height of brushes with different chain length dispersity and heights. (c) Averaged polymer (number) density profiles profile versus distance from the grafting plane.

The height of a polymer brush (*H*) is one of the most accessible properties to characterize polymer brush layers. Here, we extract the brush height (*H*) from polymer (number) density profiles as twice the first moment of the density profile in line with previous work^44,70−72^
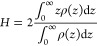
6where ρ(*z*) is the polymer number density, and *z* the distance from the grafting plane. We observe that the height of brushes increases as the dispersity increases (Figure 1b). The fully ionized brush increases from 44.9σ to 80.8σ. Hence, chain length dispersity causes large variations in the brush height.

To shed some light on these variations, we also display the polymer number density profiles of the grafted chains in the different brushes. These profiles are presented in Figure 1c. The density profiles of the monodisperse brush (*Đ* = 1.0) and the polydisperse brushes (*Đ* ≥ 1.2) show some striking differences: Monodisperse brushes have a concave profile, while the polydisperse brushes have a convex profile. These convex profiles indicate a degree of stratification in the brush, and such a change in profiles has previously been observed in simulations of disperse neutral brushes^73^ as well as in self-consistent field simulations.^74^

### Electroresponse of Fully Ionized Brushes Change Is Limited

**Figure 2:**
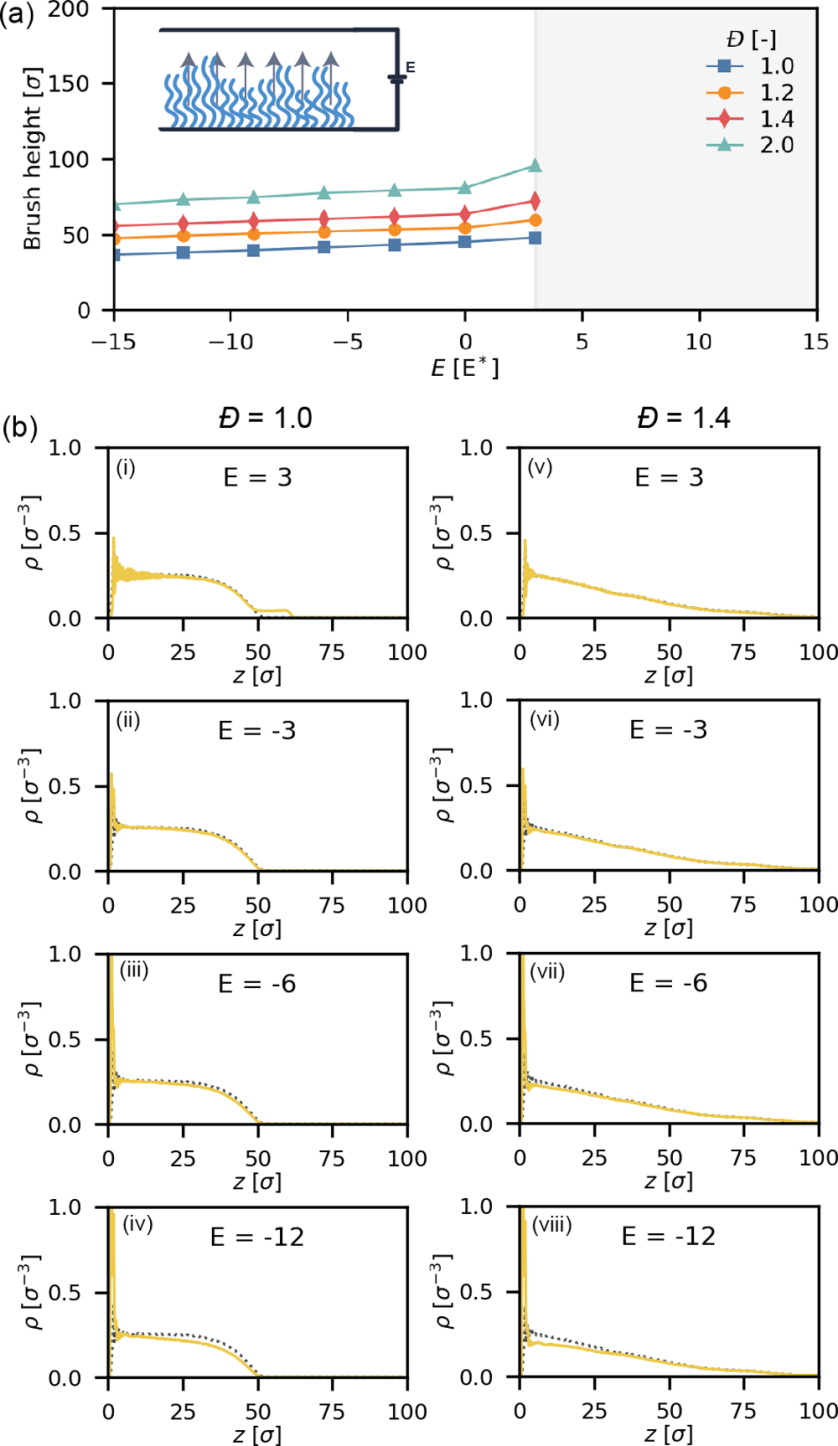
Electroresponse of fully ionized polyelectrolyte brushes with a grafting density of 0.1σ^–2^ under salt-free conditions. (a) Height of fully ionized polyelectrolyte brushes under different applied fields (*f* = 1.00). Inset: Schematic of the simulation setup. (b) Density profiles of selected fully ionized brushes under an applied field (yellow, solid line) compared to brushes at equilibrium without a field (gray, dotted line).

We study the electroresponse of the disperse, fully ionized polyelectrolyte brushes (*f* = 1.00) by applying an electric field across our coating as shown in Figure 2a. As seen previously for unperturbed conditions, the height of the brushes increases with the dispersity and this trend holds for all field strengths we evaluated. The response of these brushes in the field is rather limited: the monodisperse brush experiences a height decrease of 8.4σ from 44.9 σ at *E* = 0 E* to 36.5 σ at *E* = −15 E*, a decrease of 18.7%. The most disperse brush (*Đ* = 2.0) decreases from 80.8σ to 69.9σ, or a decrease of only 10.9 σ (−13.5%). Hence, for fully ionized brushes, chain length dispersity reduces the electroresponse of the brush for the collapse transition.

We observe a partial collapse of the brush upon introducing a negative electric field, and a partial stretching of the brush in a positive field. To illustrate this transition, we present some density profiles for selected field strengths in Figure 2b(i–iv) for *Đ* = 1.0 and in Figure 2b(v–vii) for *Đ* = 1.4. As the electric field changes in strength, slight deviations from the equilibrium profile can be observed. Specifically, the upper region of the monodisperse brush slightly decreases in density as the field strength increases to more negative values (Figure 1b). Similarly, the denser regions in the disperse brush in the disperse brush between ≈5σ and ≈30σ decrease in density (Figure 1b). Interestingly, the density of the upper region of the brush (*z* ≥ 40σ) seems to remain unaffected by the applied field. However, this change in density profile is small and only noticeable at high field strengths.

On the other hand, when we introduce a positive electric field, the response of the brush follows a different trend. When going from *E* = 0 E* to *E* = 3E*, the monodisperse brush increases in height from 44.9σ to 47.9σ (+6.7%), while the most disperse brush increases from 80.8σ to 95.6σ (+18.3%). So under these conditions, disperse brushes are more responsive to external fields. We note that fields stronger than *E* = 3 E* are not shown in Figure 1 as such fields result in a numerical instability in the simulation for disperse brushes. The combined force of multiple charged monomers on the same chain leads to overstressed bonds in the brush.

### Introducing Neutral Monomers Increases the Electroresponse

While fully ionized brushes show some response to electric fields, the height change is limited to less than 20% for the cases we evaluated. The limited magnitudes of response may limit the application of these brush coatings. Since the grafted charge in the brush affects the field strengths needed to obtain complete collapse or stretching,^43^ we study brushes with a lower degree of ionization as these should prove to be more responsive.

**Figure 3:**
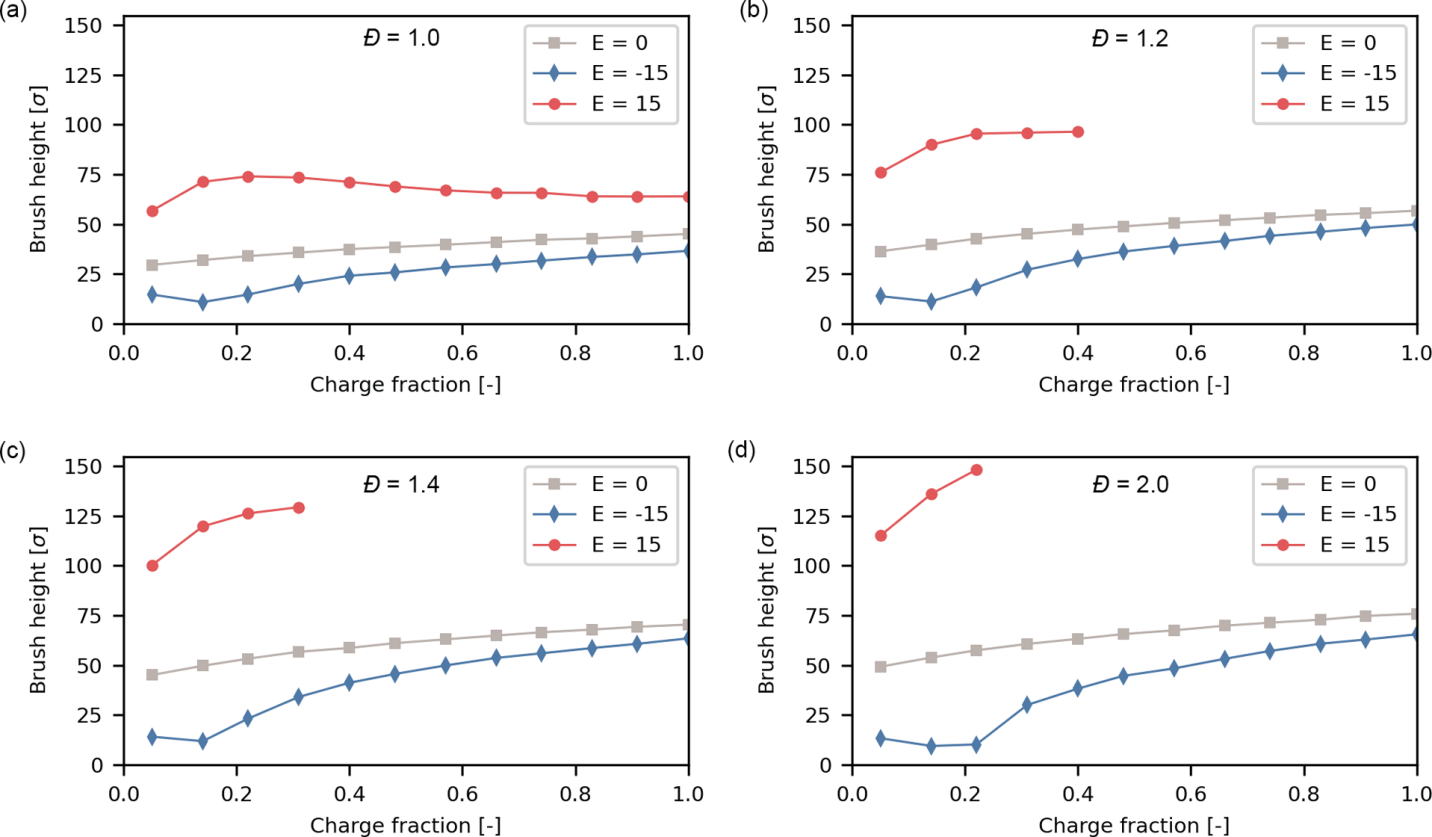
Brush height for polyelectrolyte brushes with a grafting density of 0.1σ^–2^ under salt-free conditions with different fractions of charged monomers. (a) *Đ* = 1.0 (b) *Đ* = 1.2 (c) *Đ* = 1.4 (d) *Đ* = 2.0. For nonzero dispersities at higher charge fractions, simulations become numerically unstable and no data is available.

We vary the fraction of charged chains in the brush systematically ranging from 5 to 100%. We study these brushes under three conditions: a strong negative field (*E* = −15 E*), no applied field (*E* = 0 E*), and a strong positive field (*E* = 15 E*). The brush heights of this systematic variation are portrayed in Figure 3. Considering a monodisperse brush (Figure 3a), the brush collapses more when the charge fraction is lowered up to a charge fraction of 14%. Additionally, we brush swells more but maximum swelling is achieved at a slightly higher charge fraction of 22%. Hence, the electroresponse is optimal when 14 to 22% of the monomers are charged.

Disperse brushes (Figure b–d) display a similar trend. The brush collapses to its lowest height at charge fractions of 14 or 22%. Upon stretching, the simulation becomes numerically unstable for larger charge fractions for similar reasons as discussed above. This limitation restricts the practical range for stretching to low charge fractions. Additionally, we observe that brushes collapse to the lowest height at charge fractions of 14% for most of the dispersities we study.

### Electroresponse of Partially Ionized Brushes

**Figure 4:**
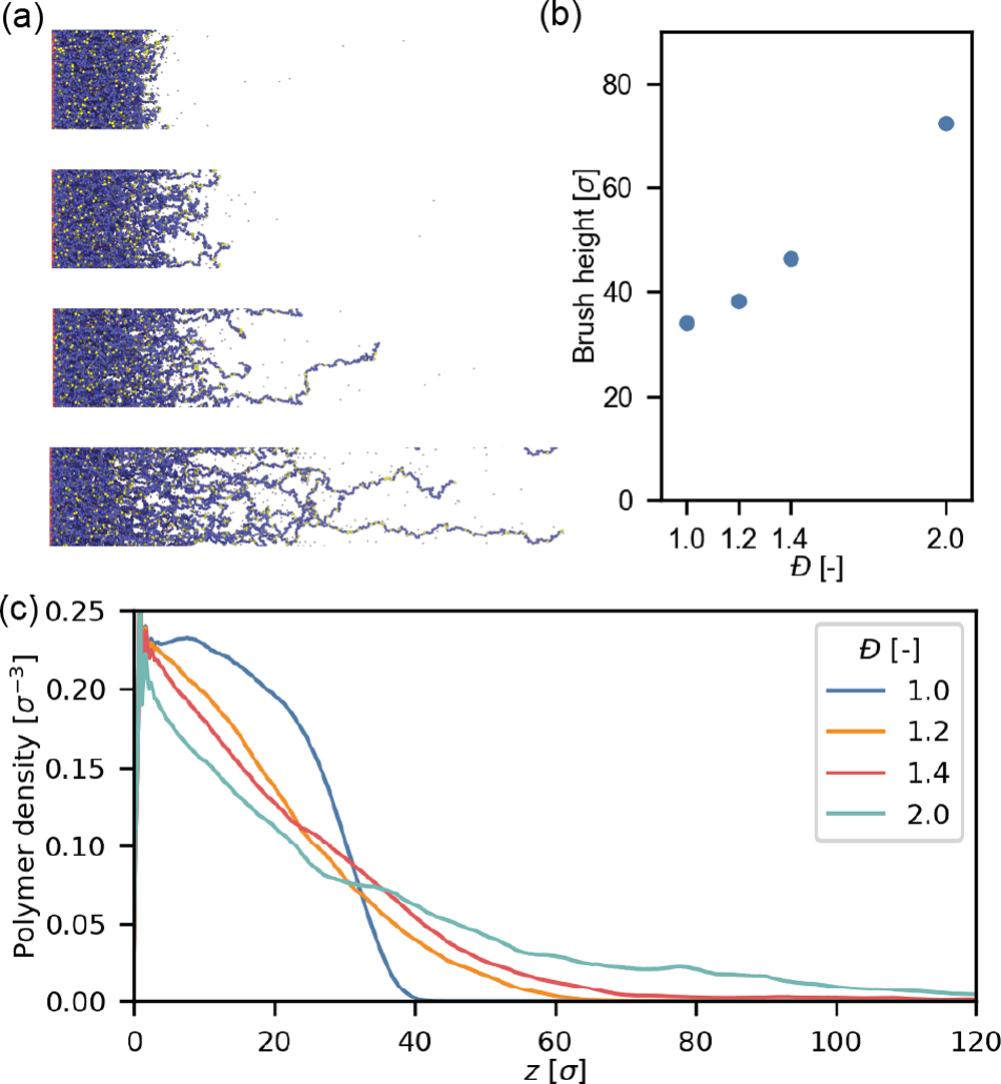
Effect of chain length dispersity on the equilibrium properties of polyelectrolyte brushes with a grafting density of 0.1σ^–2^ and a charge fraction ⟨*f*⟩ = 0.14. (a) Snapshots of the coarse-grained model. (b) Equilibrium height of brushes with different chain length dispersity and heights. (c) Averaged polymer (number) density profiles profile versus distance from the grafting plane.

The electroresponse is enhanced in partially ionized brushes and the collapse is strongest at 14% charged monomers, therefore, we study this system in more detail. The partially ionized brushes have a lower height than the fully ionized brushes (compare Figures 1b and 4b). This difference can be explained by an increased number of counterions in the fully charged brush compared to the partially charged one, which is in line with previously reported scalings between charge fraction and brush height.^75^ Additionally, the height of both partially increases in a similar manner as the fully charged brush as the dispersity increases (Figure 4b). For partially ionized brushes, the height of the monodisperse brush is 34.0σ, while the brush with *Đ* = 2.0 has a height more than twice as large with 72.4σ. Hence, chain length dispersity causes large variations in both partially and fully charged brushes.

**Figure 5:**
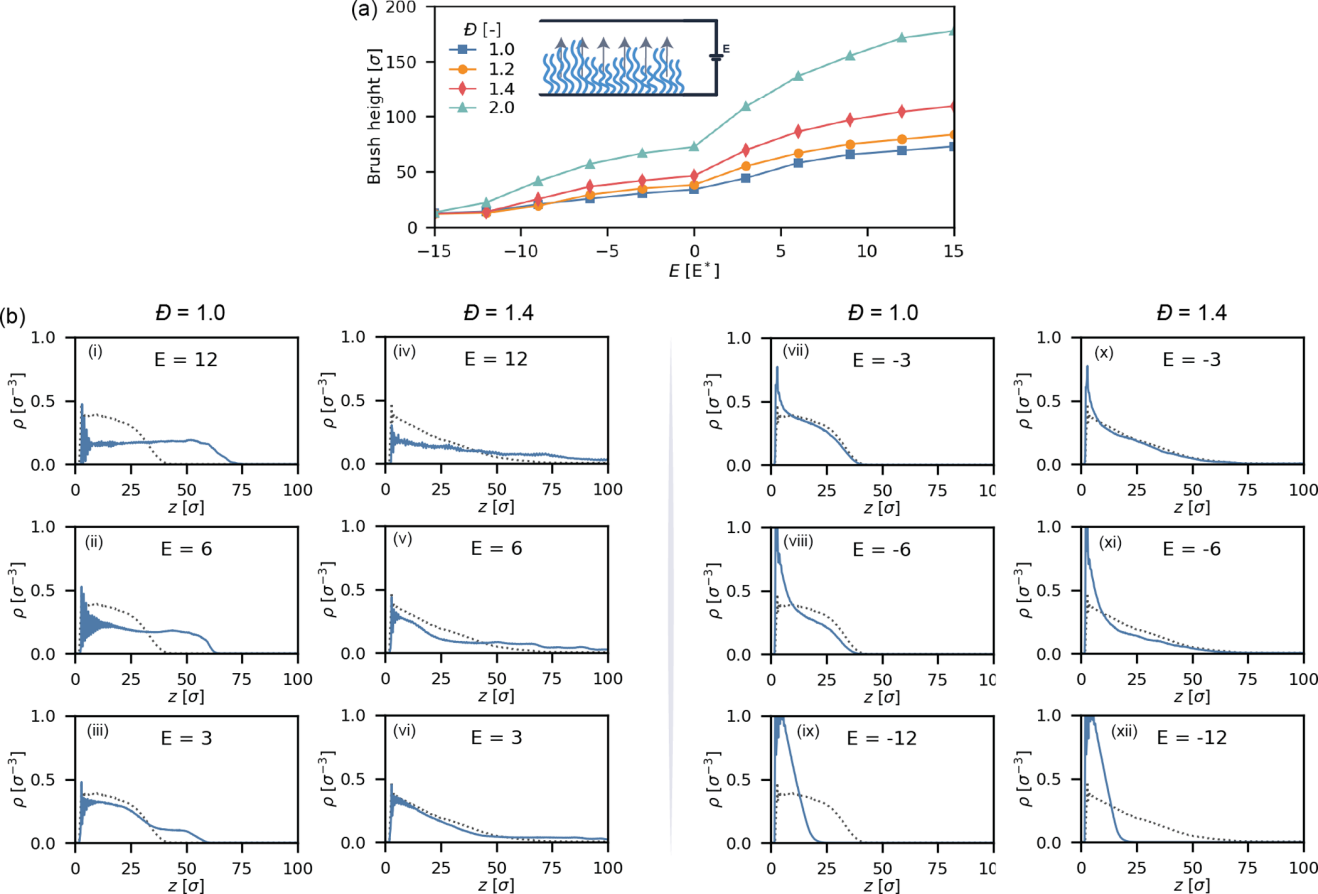
Electroresponse of partially ionized polyelectrolyte brushes with a grafting density of 0.1σ^–2^ under salt-free conditions. (a) Height of partially ionized polyelectrolyte brushes under different applied fields (*f* = 0.14). Inset: Schematic of the simulation setup. (b) Density profiles of selected fully ionized brushes under an applied field (yellow, solid line) compared to brushes at equilibrium without a field (gray, dotted line).

When the charge fraction is lowered to 14%, the response increases in magnitude. We present these response characteristics in Figure 5a. The brush height response shows several features worth noting. First, under negative fields with *E* = −15 E*, all systems collapse to roughly the same height: 12.7σ (*Đ* = 1.0), 12.0σ (*Đ* = 1.2), 12.4σ (*Đ* = 1.4), and 13.4σ (*Đ* = 2.0). So under these fully collapsed conditions, the amount of polymer in the brush seems to dominate the brush height more than the chain length dispersity. Second and as a logical consequence of the first point, this similar collapse height means that polydisperse brushes show a stronger height reduction than monodisperse brushes both in absolute and relative terms: brushes with *Đ* = 2.0 experience a height change of −59.0σ (−81.5%) compared to −21.3σ (−62.6%) for the monodisperse brush. Finally, the height under positive electric fields is also stronger for polydisperse brushes than for monodisperse brushes. Polydisperse brushes with *Đ* = 2.0 have a height increase of 105.2σ (+145.3%), while monodisperse brushes have a height increase of 38.9σ (+114.4%). Therefore, polydisperse, partially ionized brushes can respond to external fields with a larger range of brush heights than monodisperse brushes.

Besides these height changes, it is instructive to consider how the density profiles of the polymer respond to the applied fields. Figure 5b(i–iii, vii–ix) shows these density profiles for the monodisperse brush (*Đ* = 1.0) and Figure 5b(iv–vi,x–xii) shows them for a polydisperse brush (*Đ* = 1.4). Comparing these different cases, we note significant qualitative similarity for the collapsed states between the monodisperse (Figure 5b(ix)) and polydisperse brush (Figure 5b(xii)). First, the stronger the negative field, the more similar the density profiles become. This convergence also explains why the different brushes collapse to similar brush heights. Second, under positive fields, the differences between the monodisperse and polydisperse brush are exaggerated: Where the monodisperse brush maintains a mostly concave profile with a near constant density in the middle of the brush (Figure 5b(i)), the polydisperse brush keeps a convex profile that stretches to higher heights with increasing field strengths (Figure 5b(iv)). Such qualitative differences are interesting to note as in experiments these may result in different interactions of these brushes with the surrounding medium.

### Short Chains Collapse First, Long Chains Stretch First

The previous sections revealed that monodisperse and polydisperse polyelectrolyte brushes restructure in electric fields. The fully ionized brushes showed a weaker response to electric fields than the partially ionized brushes, even though the former contains more charges on which the electric field can act. Additionally, the collapse of fully ionized brushes is reduced if polydispersity is increased, while for partially ionized brushes it increases. What can explain these apparent discrepancies?

**Figure 6:**
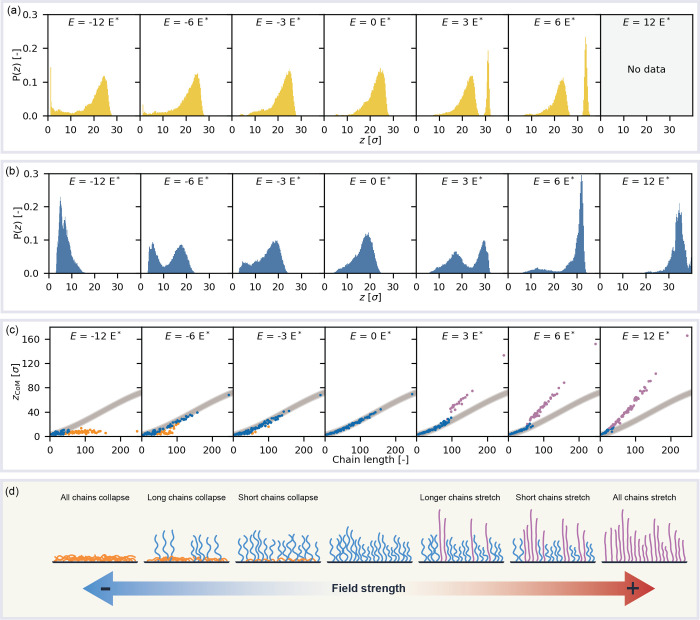
Electroresponse of individual chains in polyelectrolyte brushes with a grafting density of 0.1σ^–2^. (a) Normalized distribution of the out-of-plane center-of-mass coordinates of individual chains in a fully ionized brush under selected applied fields (*f* = 1.00). (b) Normalized distribution of the out-of-plane center-of-mass coordinates of individual chains in a partially ionized brush under selected applied fields (*f* = 0.14). (c) Out-of-plane center-of-mass coordinates of individual chains in a partially ionized brush (*f* = 0.14, *Đ* = 1.4) compared to their contour length under various applied fields. The gray band indicates the unperturbed brush as a reference. Blue points fall within the unperturbed model; purple data points are stretched compared to the unperturbed situation; orange point indicate collapsed points. (d) Schematic overview of the stratified electroresponse of disperse polyelectrolyte brushes.

To explain this discrepancy, we investigate the response of individual chains the brush compared to the collective response of the complete brush. First, we observe the behavior of chains in monodisperse brushes. Figure 6 shows the distribution of the center of mass of individual chains in the brush for fully ionized brushes (Figure 6a) and partially ionized brushes (Figure 6b). For the partially ionized brush in negative fields, we observe that the center of mass distribution gradually shifts density from its equilibrium distribution around 19σ to a new mode around 5σ. For the fully ionized brush, this shift is less pronounced. In positive fields, a similar shift in center of mass density can be seen with a new mode appearing around 31σ. These profiles show an important characteristic of brush switching: Brushes respond to electric fields by partitioning into two populations of which one responds to the stimulus and collapses or stretches and the other remains mostly unaffected. This partial response effect has been pointed out in the literature previously.^32,34,42,43^

The bimodal response to electric fields has profound implications for the response of disperse polyelectrolyte brushes. Brushes respond to fields in a chain by chain fashion because it is energetically favorable to deform a limited number of chains significantly compared to all chains partially. Since polydisperse brushes contain chains of different lengths, chains have different contributions to the free energy of the system and a differentiated response based on chain length can be expected.

We study these effects of chain length on the collapse of individual chains using similar metrics as in Figure 6a,b. For all chains in the brush, we compute its average center of mass, which provides us with the capacity to correlate the chain length to its center of mass. We can than plot these data points in a scatter plot; in Figure 6c we present this data for the polydisperse, partially ionized brush with *Đ* = 1.4. We plot the case where *E* = 0 E* which in the middle panel. In what follows, we guide the reader in interpreting the data visualization by describing the construction of the other elements in the visualization. First, we fit a third-order polynomial to the scatter data of the brushes for *E* = 0 E*. We use standard deviation of the residuals as the standard deviation of this model. We then visualize the model values and indicate 3 standard deviations from the model as a gray band in the plot. Finally, we plot the average center of mass of each chain and color code the data points such that all points more than 4 standard deviations from the model prediction are colored purple (for stretched chains) or orange (for collapsed chains).

Figure 6c reveals an intriguing effect in the electroresponse of polydisperse brushes: short chains collapse first, while long chains stretch first as we schematically depict in Figure 6d. For instance, if we consider the collapse transition (*E* < 0 E*), chains longer than approximately 110 monomers only significantly deviate from their unperturbed conditions when the field strength increases to *E* = −9 E*. As the magnitude of the field strength increases, the length of the chain that reorganize becomes longer and longer until all chains collapse at *E* ≤ – 12 E*. Hence, as a result of this bimodal response behavior, shorter chains collapse preferentially over longer chains.

Interestingly, the opposite effect is observed in the stretching transition. For small electric fields, only longer chains stretch and shorter chains do not significantly deviate from their equilibrium center of mass. As the field strength increases, the chain lengths of the responding population start to become smaller until nearly all chains attain a deformed conformation at fields larger than 12 E*. So in polydisperse brushes longer chains stretch first.

## Discussion

Our simulations reveal three key observations on the electroresponse of disperse polyelectrolyte brushes. First, lowering the fraction of charged monomers in the brush enhances the electroresponse. Second, increasing the dispersity increases the electroresponse. And third, the chains in a disperse brush show a stratified response where short chains collapse first and long chains stretch first.

It may seem counterintuitive that decreasing the charge fraction in a brush increases its electroresponse. Should fewer charges not experience a weaker effect? This counterintuitive effect can be explained by considering that the brush screens the electric field in the system. In order to screen an electric field, polyions (or counterions) localize near the electrode to form a screening layer. The strength of this screening layer depends on the charge it contains: the more charges, the more screening. Hence, if a fully charged polyelectrolyte chain collapses, all segments in the chain contribute a charge to this screening layer, and few chains are required to achieve this screening. On the other hand, for partially charged brushes, more chains need to collapse in order to screen the electric field. Therefore, more partially charged chains collapse than fully charged chains at identical field strengths. A similar reasoning explains the enhanced stretching of partially charged chains. The reduction in charge cannot be continued indefinitely. After all, neutral chains do not respond to electric fields. Therefore, the system behaves nonmonotonically upon a variation of the charge fraction, where for our system we find an optimal response around 14% charged monomers.

Dispersity adds to the electoresponse of polyelectrolyte brushes. Disperse brushes contain longer chains that reach to higher heights, so the same amount of polymer has a higher average height. This increased starting height also means that a fully collapsed brush–where the polymer forms a nearly space-filling layer near the electrode–has a stronger height reduction. Additionally, in stretching fields, the longer chains can reach to much higher heights, allowing a much higher brush height. In our simulations, we limit the dispersity in the simulations to *Đ* ≤ 2 in line with values of dispersity that are commmonly reported. Larger dispersities typically indicate poor control over the polymerization, but there are no fundamental reasons why larger dispersities would be impossible. It would be interesting to see experimental work where dispersity is treated as an additional design parameter.

In the simulations, we observe a stratification in the electoresponse based on chain length. Shorter chains collapse first, because of both topological reasons—they are closer to the electrode—and entropic reasons—their collapse is accompanied by a lower loss of entropy. Longer chains stretch first, because stretching a long chain has a lower entropic penalty than longer chains since their relative extension will be lower initially.

Our simulations have explored the effect two parameters on the electroresponse of polyelectrolyte brushes, but one can envision various other brush parameters that could be varied. These parameters include the distribution of charge along the polymer backbone as well as the grafting density. In previous work,^43^ we explored the effect of these parameters in monodisperse brushes. These brushes maintain their electroresponse and their full response is dominated by the grafted charge (i.e., the product of the charge per chain and the grafting density). To combine these effects, one could implement a different method to construct the polyelectrolyte brushes in the simulation, for instance by doing an in silico polymerization based on reactivities of different monomers and (optionally) termination rates. Brushes of these polymers would then be more representative of brushes grown in laboratories at the expense of being able to study the effect of specific brush parameters on the brush response.

Finally, we remark that our simulations have been performed in salt-free conditions for equilibrium conditions. In many practical situations, brushes are exposed to conditions that contain traces of salt. This salt may contribute to screening the electric field and hence weaken the electroresponse of the brush. In ongoing work, we are currently studying the effect of this external influence in more detail. Additionally, our simulations probe the equilibrium response of the brushes, yet the dynamics of the electroresponse are also of particular interest for applications. These dynamics determine the response time of brushes and the frequency at which these systems can be switched. While we hypothesize that brushes with longer chains need longer to switch, a detailed discussion of the response dynamics cannot be done based on our simulations and warrant a detailed study in and of itself.

## Conclusions and Practical Implications

In this work, we present two counterintuitive strategies to enhance the electroresponse of polyelectrolyte brushes: decreasing the charge fraction and increasing the dispersity of the chains in the brush. These strategies follow from coarse-grained molecular dynamics simulations that reveal three key trends. All of which can have implications for the use of polyelectrolyte brushes in electro-responsive applications. First, the electroresponse of polyelectrolyte brushes increases when the polymer contains a smaller charge. From a practical perspective, this means that the electroresponse of polyelectrolyte brushes can be increased if one decreases the charge on the polymers. A strategy to achieve this would be to synthesize a copolymer brush of monomers without a charge and monomers with fixed charges (e.g., sulfonate or quarternized ammonia moieties). These coatings could be fabricated using postmodification strategies of neutral brushes like the quarternization of PDMAEMA^76^ or via copolymerization of charged and neutral monomers.^77,78^ Both strategies present unique opportunities as the chemistry of the neutral monomers can be adjusted to the desired application. Second, brushes with a higher dispersity show a stronger collapse than monodisperse brushes. Progress in polymer brush synthesis allows for increasingly well-controlled chain length dispersities in brushes,^18^ so this parameter can be used as a design feature to tune the behavior of the system. Such control would be useful in applications where brushes form a physical barrier to prevent the migration of specific molecular species or fluids through membranes or nanochannels. And finally, in disperse brushes, short chains collapse first, while long chains stretch first. In many living polymerizations, the chain ends of brushes can be postmodified with a functional group, for instance a polypeptide, DNA, or antibody. If an application relies on the expression of the moiety at the surface of the brush, this stratified effect can have significant implications. We envision that our results will enable the development of new electroresponsive technologies that use polyelectrolyte brushes to introduce additional features to functional surfaces.

## Supporting Information Available

Root-mean squared deviation during production run (PDF)
