# Supplementary material for: Molecular Design Strategies to Enhance the Electroresponse of Polyelectrolyte Brushes: Effects of Charge Fraction and Chain Length Dispersity
Source: Macromolecules. 2025 Jan 23;58(3):1185–95. doi: 10.1021/acs.macromol.4c02579 (PMC11823628; doi:10.1021/acs.macromol.4c02579)
Supplement: Supplementary file 1 — ma4c02579_si_001.pdf [file ma4c02579_si_001.pdf]

Supplementary Information: Molecular design strategies to enhance the electroresponse of polyelectrolyte brushes: effects of charge fraction and chain length dispersity

Leon A. Smook* and Sissi de Beer*

l.a.smook@utwente.nl; s.j.a.debeer@utwente.nl

Department of Molecules & Materials, MESA+ Institute, University of Twente, P.O. Box 217, 7500 AE Enschede, The Netherlands

Contents

S1 Root-mean squared deviation during production run **S2**

S1.1 Negative fields S2

S1.2 No fields S3

S1.3 Positive fields S3

S1 Root-mean squared deviation during production run

To confirm that our simulations have reached equilibrium, we observe the root-mean-square deviation of the polymer over the production run. We observe that the RMSD value quickly settles at a stable value. This indicates that the system has equilibrated. In this supporting information section, we show several representative RMSD curves for different charge fractions at strong collapsing fields ($E = -15\ E^*$), no field ($E = 0\ E^*$), and strong stretching fields where the simulations remain numerically table ($E = 15\ E^*$).

S1.1 Negative fields

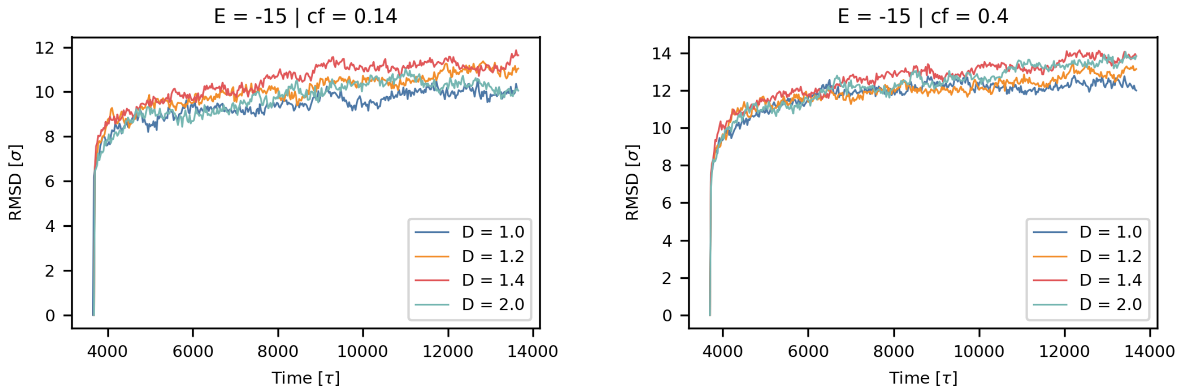

Figure S1: RMSD curves for selected brushes at strong negative fields ($E = -15E^*$) with a charge fraction of 0.14 (left), 0.4 (right).

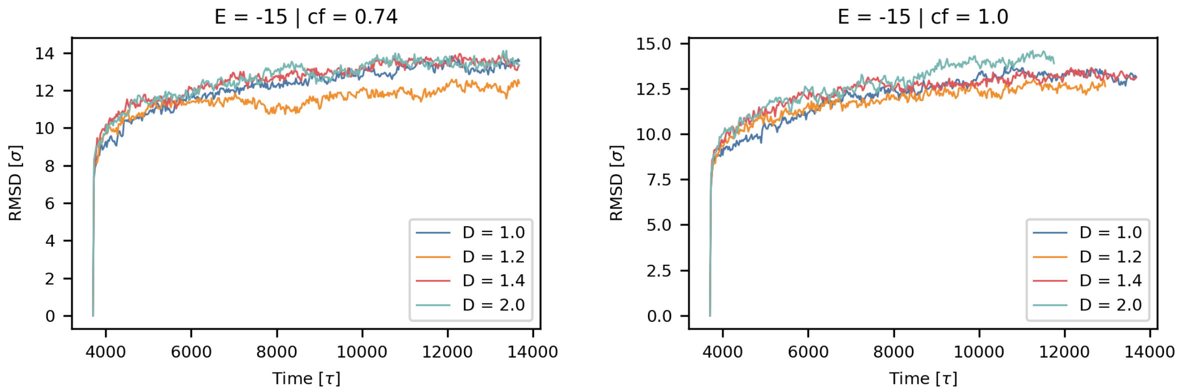

Figure S2: RMSD curves for selected brushes at strong negative fields ($E = -15E^*$) with a charge fraction of 0.74 (left), 1.0 (right).

S1.2 No fields

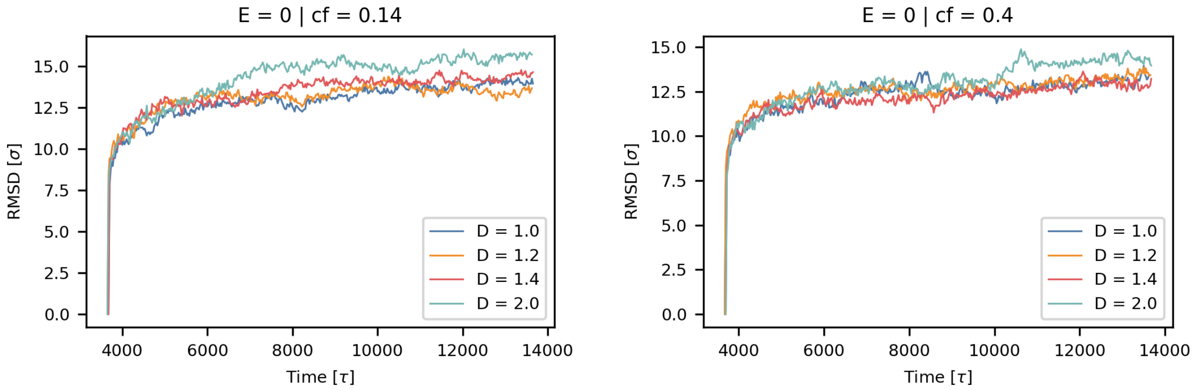

Figure S3: RMSD curves for selected brushes in the absence of electric fields ($E = 0E^{*}$) with a charge fraction of 0.14 (left), 0.4 (right).

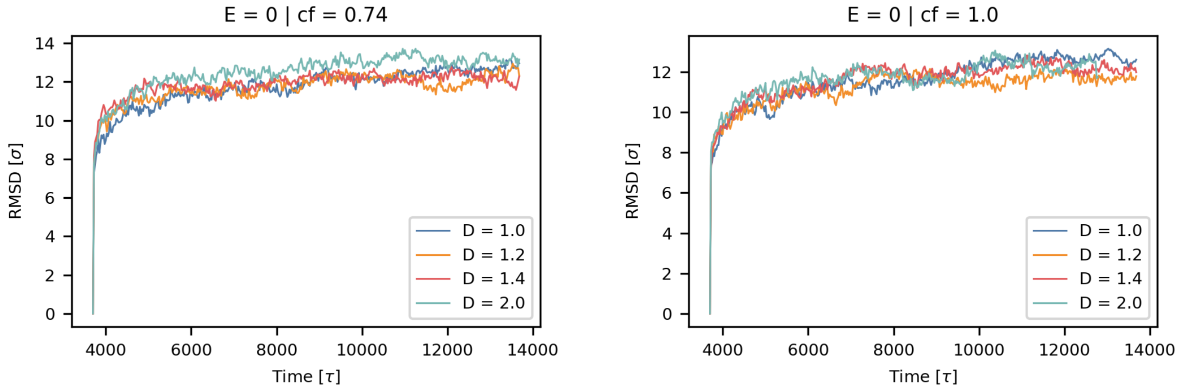

Figure S4: RMSD curves for selected brushes in the absence of electric fields ($E = 0E^{*}$) with a charge fraction of 0.74 (left), 1.0 (right).

S1.3 Positive fields

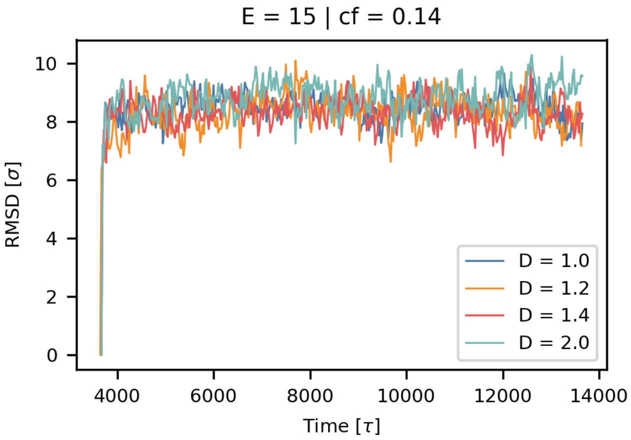

Figure S5: RMSD curves for selected brushes at strong negative fields ($E = 15E^{*}$) with a charge fraction of 0.14.
